# Axial Spondylometaphyseal Dysplasia Is Caused by *C21orf2* Mutations

**DOI:** 10.1371/journal.pone.0150555

**Published:** 2016-03-14

**Authors:** Zheng Wang, Aritoshi Iida, Noriko Miyake, Koji M. Nishiguchi, Kosuke Fujita, Toru Nakazawa, Abdulrahman Alswaid, Mohammed A. Albalwi, Ok-Hwa Kim, Tae-Joon Cho, Gye-Yeon Lim, Bertrand Isidor, Albert David, Cecilie F. Rustad, Else Merckoll, Jostein Westvik, Eva-Lena Stattin, Giedre Grigelioniene, Ikuyo Kou, Masahiro Nakajima, Hirohumi Ohashi, Sarah Smithson, Naomichi Matsumoto, Gen Nishimura, Shiro Ikegawa

**Affiliations:** 1 Laboratory of Bone and Joint Diseases, RIKEN Center for Integrative Medical Sciences, Tokyo, 108–8639, Japan; 2 McKusick-Zhang Center for Genetic Medicine and State Key Laboratory of Medical Molecular Biology, Institute of Basic Medical Sciences, Chinese Academy of Medical Sciences & Peking Union Medical College, Beijing, 100005, China; 3 Department of Human Genetics, Yokohama City University Graduate School of Medicine, Yokohama, 236–0004, Japan; 4 Department of Advanced Ophthalmic Medicine, Tohoku University Graduate School of Medicine, Sendai, 980–8574, Japan; 5 Department of Retinal Disease Control, Tohoku University Graduate School of Medicine, Sendai, 980–8574, Japan; 6 Department of Opthalmology, Tohoku University Graduate School of Medicine, Sendai, 980–8574, Japan; 7 Department of Pediatrics, King Abdulaziz Medical City for National Guard Health Affairs, Riyadh, 22490, Saudi Arabia; 8 Department of Pathology and Laboratory Medicine, King Abdulaziz Medical City, National Guard Health Affairs, Riyadh, 22490, Saudi Arabia; 9 Department of Radiology, Woorisoa Children's Hospital, Seoul, 08291, Republic of Korea; 10 Department of Orthopaedic Surgery, Seoul National University College of Medicine, Seoul, 03080, Republic of Korea; 11 Department of Radiology, St. Mary’s Hospital, The Catholic University, Seoul, 07345, Republic of Korea; 12 CHU Nantes, Service de Génétique Médicale and INSERM, UMR-S 957, Nantes, 44093, France; 13 Department of Medical Genetics, Section for Clinical Genetics, Oslo University Hospital, Oslo, 0424, Norway; 14 Department of Radiology, Oslo University Hospital, Oslo, 0424, Norway; 15 Department of Medical Biosciences, Medical and Clinical Genetics, Umeå University, Umeå, 90187, Sweden; 16 Department of Clinical Genetics and Department of Molecular Medicine and Surgery, Karolinska Institutet, Karolinska University Hospital, Stockholm, 17176, Sweden; 17 Division of Medical Genetics, Saitama Children’s Medical Center, Saitama, 339–8551, Japan; 18 Department of Clinical Genetics, St. Michaels Hospital, Bristol, BS2 8EG, United Kingdom; 19 Department of Pediatric Imaging, Tokyo Metropolitan Children's Medical Center, Fuchu, 183–8561, Japan; Innsbruck Medical University, AUSTRIA

## Abstract

Axial spondylometaphyseal dysplasia (axial SMD) is an autosomal recessive disease characterized by dysplasia of axial skeleton and retinal dystrophy. We conducted whole exome sequencing and identified *C21orf2* (chromosome 21 open reading frame 2) as a disease gene for axial SMD. *C21orf2* mutations have been recently found to cause isolated retinal degeneration and Jeune syndrome. We found a total of five biallelic *C21orf2* mutations in six families out of nine: three missense and two splicing mutations in patients with various ethnic backgrounds. The pathogenic effects of the splicing (splice-site and branch-point) mutations were confirmed on RNA level, which showed complex patterns of abnormal splicing. *C21orf2* mutations presented with a wide range of skeletal phenotypes, including cupped and flared anterior ends of ribs, lacy ilia and metaphyseal dysplasia of proximal femora. Analysis of patients without *C21orf2* mutation indicated genetic heterogeneity of axial SMD. Functional data in chondrocyte suggest *C21orf2* is implicated in cartilage differentiation. C21orf2 protein was localized to the connecting cilium of the cone and rod photoreceptors, confirming its significance in retinal function. Our study indicates that axial SMD is a member of a unique group of ciliopathy affecting skeleton and retina.

## Introduction

Spondylometaphyseal dysplasia (SMD) is one of the currently defined 40 groups of genetic skeletal disorders (group 12) [1]. It refers to abnormal development involving both spine and metaphyses of long bones. Axial SMD (MIM 602271) is a clinical subtype of SMD, in which mainly axial skeleton and retina are affected [2]. The skeletal manifestations of axial SMD include dysplasia of the ribs, vertebral bodies, ilia, and proximal femora. Axial SMD patients also show impaired visual acuity at early ages, and are usually diagnosed with retinitis pigmentosa during childhood. The presence of equally affected sibling pairs of both genders, and parental consanguinity in some affected families [2–4], strongly suggests autosomal recessive inheritance of axial SMD. However, the disease-causing gene of axial SMD has not been identified, and its molecular pathogenic mechanism is unknown.

Here, by performing whole exome sequencing on axial SMD patients, we identified *C21orf2* as a disease gene for axial SMD. In parallel to our work, *C21orf2* mutations have recently been identified in patients with rod-cone dystrophy and posterior staphyloma without skeletal features[5] and in patients with Jeune syndrome[6], which is also known as asphyxiating thoracic dysplasia (OMIM 263510). The skeletal phenotypes of axial SMD are very diverse even between individuals with the same *C21orf2* mutations. We found evidence for genetic heterogeneity of axial SMD. Our functional data in chondrocyte suggest *C21orf2* is implicated in cartilage differentiation. Our *C21orf2* expression analysis in retina suggests that axial SMD is a ciliopathy.

## Results and Discussion

### Patients and their clinical features

**Fig 1:**
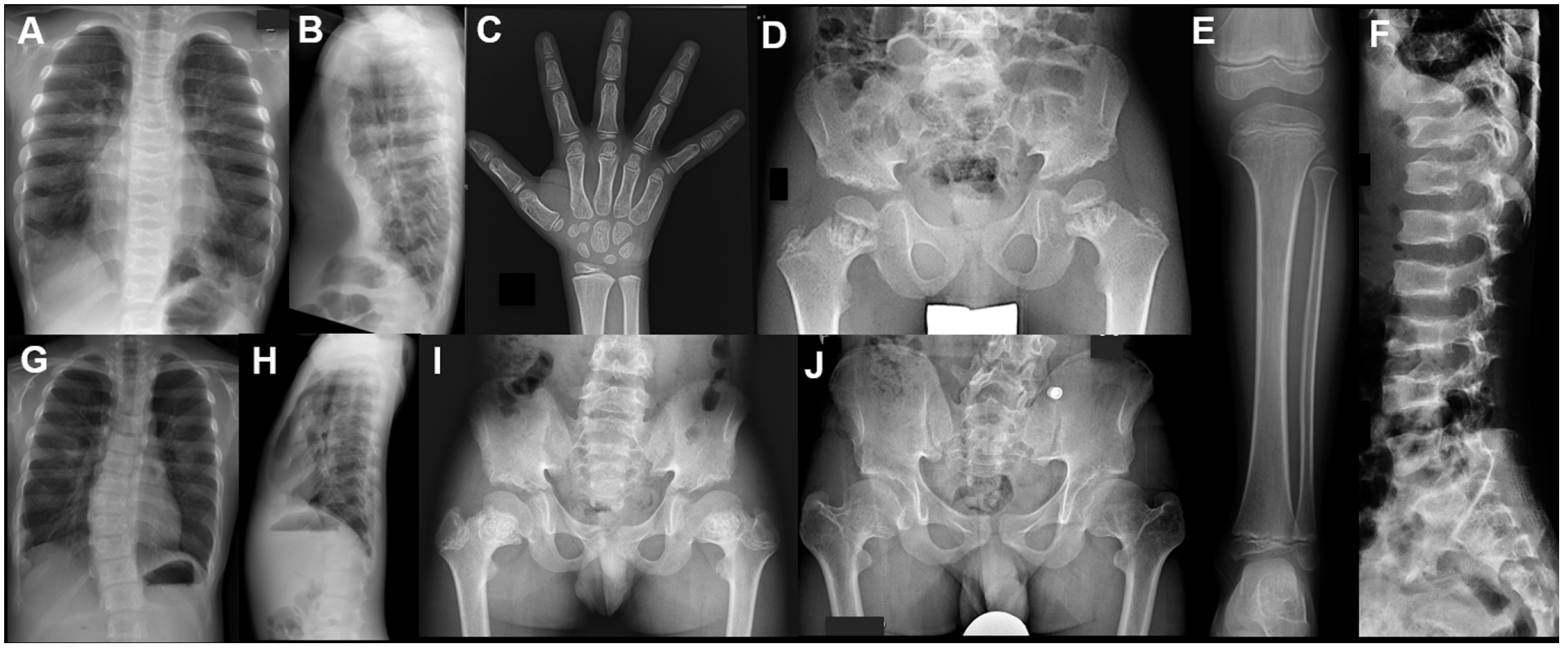
Radiographic features of axial SMD. (A-F) P7 at age 6 years. Note narrow thorax, short ribs with cupped anterior ends, mildly serrated iliac wings, short ilia, metaphyseal irregularities and shortening of the proximal femora, and mild platyspondyly. Metaphyses of knee and ankle are normal. Hands are normal. G-I) P5 at age 10 years. Narrow thorax with short ribs, mildly serrated iliac wings, short ilia, and metaphyseal irregularities and shortening of the proximal femora. He had mild scoliosis, but platyspondyly is not evident. J) P5 at age 14 years. Note progressive shortening and varus deformity of the proximal femora.

Thirteen patients with axial SMD from nine families (Table 1) were included in this study. Written informed consents were obtained from all the participants. Families F1–F6 have been described previously [2–4]. Key clinical features of all patients, including updates of the patients in F1–F6, are summarized in Table 1. The common clinical findings among the patients include 1) mild postnatal growth failure, 2) severe thoracic deformity (S1 Fig), 3) impaired visual acuity and retinal dystrophy (diagnosed as retinitis pigmentosa or cone-rod dystrophy). In all patients, impaired visual acuity came to medical attention in early life, and retinal function deteriorated rapidly. Thoracic hypoplasia, due to severe shortening of the ribs, was also observed in all patients. The remarkably narrow and long chest might restrict the expansion and development of lung, and therefore could be the cause of neonatal respiratory problems and susceptibility to airway infection. The radiological features of the patients included cupped and flared anterior ends of ribs, lacy ilia (serrated iliac crests), and metaphyseal dysplasia of proximal femora (Fig 1). Mild platyspondyly was common, but the height of vertebral bodies could sometimes be normal. The proximal femoral metaphyses were irregular (enchondroma-like). Shortening of the femoral neck was often progressive, resulting mild coxa vara in older patients. Metaphyseal dysplasia was rarely seen in other long tubular bones. None of the patients had brain or kidney complications, or polydactyly.

**Table 1:** Clinical, radiographic and genetic findings of the axial SMD subjects.

Patient ID	P1-1	P1-2	P2	P3-1	P3-2	P4	P5	P6	P7	P8-1	P8-2	P8-3	P9
Family ID	F1	F1	F2	F3	F3	F4	F5	F6	F7	F8	F8	F8	F9
**C21orf2 mutation**	+	+	–	–	–	–	+	+	+	+	+	+	+
**General information**													
ethnicity	Saudi Arabian	Saudi Arabian	Japanese	Korean	Korean	Japanese	Korean	French	Saudi Arabian	Turkish	Turkish	Turkish	Swedish
consanguinity of parents	+	+	–	–	–	–	–	+	+	+	+	+	probably +
sex	F	M	F	M	F	M	M	M	M	M	F	F	F
age (at last visit) [yr]	4.6	7	0.9	23	15	12	15	14.8	9	9	37	30	28
birth length [cm]	51	51	47.6	NA	NA	48.5	49	51.5	NA	50	NA	NA	49
birth weight [g]	3,440	3,480	NA	3,000	2,880	3,052	2,350	3,850	NA	3,600	NA	NA	3,400
**Clinical feature**													
growth delay/short stature	+	+	+	+	+	+	+	+	+	+	+	+	+
narrow thorax	+	+	+	+	+	+	+	+	+	+	+/–	+/–	+
short trunk	–	–	–	+	+	+	+	–	–	–	–	–	–
scoliosis	–	–	–	–	–	+	+	–	–	–	–	–	+
upper limb rhizomelic shortening	+	+	+	+	+	+	+	+	–	–	NA	NA	+
respiratory disturbance at birth	–	–	+	–	–	+	–	–	+	+	NA	NA	–
retinal dystrophy	CRD	CRD	RP	RP	RP	RP	RP	CRD	RP	RP	RP	RP	RP
other eye problems				optic atrophy, nystagmus	optic atrophy, nystagmus	cataract, nystagmus		photophobia		ptosis			
**Radiological feature**													
platyspondyly	–	–	–	+	+	+	–	–	+	+	+	+	–
short rib	+	+	+	+	+	+	+	+	+	+	NA	NA	+
cupped anterior end of rib	+	+	+	+	+	+	+	+	+	+	NA	NA	–
lacy ilia	+	+	–	+	+	+	+	+	+	–	NA	NA	NA
coxa vara	–	–	–	+	+	+	+	–	–	–	+	+	+
flattening of femoral head	+	+	NA*	+	+	–	–	+	+	+	+	+	+
metaphyseal dysplasia of													
–*proximal femur*	+	+	+	+	+	+	+	+	+	+	–	–	–
–*other long bones*	–	–	–	–	–	–	+	NA	–	–	NA	NA	NA
–*metacarpal*	+	+	–	–	–	–	+	–	–	+	NA	NA	–

NA: not available or assessed, RP: retinitis pigmentosa, CRD: cone-rod dystrophy.

*epiphyses are not ossified.

### Whole exome sequencing and mutation detection

We performed whole exome sequencing on ten patients from eight families (F1–F8). The mean coverage depths for reads ranged from 75.7× to 218.8× among the sequenced individuals; in general, ~90% of targeted bases in each exome had sufficient coverage (20× coverage or more) and quality for variant calling (S1 Table). In five of the eight families, homozygous (in F1, F6, F7 and F8) or compound heterozygous (in F5) variations were found on *C21orf2* (chromosome 21 open reading frame 2) based on the autosomal recessive model. All variations were confirmed by using Sanger sequencing. In F9, we directly performed Sanger sequencing for all exons and surrounding intronic regions of *C21orf2*, and found a homozygous mutation.

In total, we found bi-allelic mutations in *C21orf2* in six out of the nine families (Table 2). The origin of each mutant allele was confirmed by checking parental DNA members. All mutations showed co-segregations among available family samples. The 12 mutant alleles were counted as five different mutations, including three exonic mutations (c.218G>C, c.319T>C and c.347C>T, S5 Fig; NM_004928), and two intronic mutations (c.545+1G>A and c.643-23A>T). Except c.545+1G>A, all detected mutations were not reported in the Human Gene Mutation Database (HGMD). c.545+1G>A has previously been reported as a causal mutation of cone-rod dystrophy [7]. The skeletal phenotype of the patient with this mutation is not described in the publication.

**Table 2:** *C21orf2* mutations in axial SMD.

Family	Mutation		
	Nucleotide change^a^	Amino acid change^b^	Location
1	c.643-23A>T	p.N215Vfs*259	Intron 6
5	c.319T>C	p.Y107H	Exon 4
	c.347C>T	p.P116L	Exon 4
6	c.545+1G>A	p.[S183*, A181Qfs*6]^c^	Intron 5
7	c.643-23A>T	p.N215Vfs*259	Intron 6
8	c.218G>C	p.R73P	Exon 4
9	c.218G>C	p.R73P	Exon 4

^a^ The nucleotide changes are shown with respect to *C21orf2* mRNA sequence (NM_004928).

^b^ The corresponding predicted amino acid changes are numbered from the initiating methionine residue.

^c^ There are various minor splicing variants (See Fig 2C).

### Characterization of *C21orf2*

*C21orf2* (OMIM: 603191) is an uncharacterized gene. *C21orf2* was reported to have four alternative transcripts (NM_004928, NM_001271440, NM_001271441, and NM_001271442) in the Reference Sequence Database (RefSeq) and a previous report [8]. NM_004928, NM_001271440 and NM_001271441 have some in-frame indels but share the same reading frame, while NM_001271442 uses a different ATG as a translation start codon. As the basis of clarifying the biological impact of the detected variations, we first validated the gene structure of *C21orf2* by performing RT-PCR and sequencing of cDNAs from various tissues and cell-lines. Besides common tissues (brain, heart, lung, liver, kidney, *etc*.), additional attention was paid to bone, cartilage and retina tissues as well as related cell lines, because they may have potential relationships with the axial SMD phenotype. A pair of primers was designed to cover the whole coding DNA sequence (CDS) of transcripts NM_004928, NM_001271440 and NM_001271441. *C21orf2* was expressed in all tissues and cell-lines tested, with a single band generated (S3 Fig). The ubiquitous expression of *C21orf2* is consistent with records in gene expression databases (FANTOM5 and MGI Gene Expression Database). Sequencing of the PCR products from various tissues including chondrocyte, mesenchymal stem cell and ligament confirmed the existence of NM_004928 and NM_001271440, which differed by three nucleotides in the beginning of exon 6, resulting in one optional serine without changing the reading frame. NM_001271441 was not found in all samples examined. Primers based on NM_001271442 specific sequence could not yield targeted amplification (data not shown); probably it does not exist in tested tissues and cell-lines. For simplicity, we describe all variations based on NM_4980. NM_4980 is a 2,233-bp mRNA, which encodes a protein containing 256 amino acids (NP_004919).

By using HomoloGene and BLAST, we found that C21orf2 protein has homologues in nearly all genome-sequenced vertebrates (S4 Fig). The alignment of C21orf2 and its orthologous proteins identified two highly conserved regions: one in the N-terminal (1–142 aa, coded by exons 1–5), and the other one in the C-terminal (214–256 aa, coded by exon 7); on contrary, the middle part of C21orf2 (143–213 aa, coded by exons 5–6) is quite variable among species (S4 Fig). In the N-terminal conserved region, a predicted mitochondria localization signal peptide, two tandem leucine-rich repeat 4 (LRR_4) domains followed by a leucine-rich repeat cap (LRRcap) domain were recognized by their characteristic motifs. Neither the C-terminal conserved region nor the variable region have any homology to known domains and proteins.

### Characteristics of *C21orf2* mutations

Three missense variations were found in this study. c.319T>C [p.Y107H] and c.347C>T [p.P116L], were found in family F5 from Korea (S5 Fig). The patient (P5) was a compound heterozygote. Both variations were not found in ESP6500, although c.319T>C has low allele frequencies in 606 unrelated Korean controls (0.082%, one heterozygous allele found) and in ExAC (0.00223%). Another missense mutation, c.218G>C [p.R73P], was found in families F8 from Turkey and F9 from Sweden. A homozygosity mapping of F8 showed *C21orf2* was in a long homozygous stretch. c.218G>C was absent in 100 Turkish control individuals, but was reported in ESP6500 and ExAC (rs140451304) with very low allele frequencies (0.0154% and 0.0334%, respectively).

The three missense variations were all located in the N-terminal conserved region; c.218G>C ([p.R73P]) was in the second LRR domain, and c.319T>C ([p.Y107H]) and c.347 C>T ([p.P116L]) were in the LRRcap domain (S4B Fig). The amino acids at those positions were highly conserved among diverse species (S4 and S5 Figs). Impacts of the missense mutations were estimated in SIFT, PolyPhen and MutationTaster. All mutations were regarded as damaging by at least one of the prediction programs. 3D-protein predictions by I-TASSER showed significant structural changes in the mutants.

Two variations outside the coding region of *C21orf2* were observed. c.643-23A>T was found in two Saudi Arabian families (F1 and F7), and was absent in all control groups, including ESP6500 and ExAC databases. c.643-23A>T was predicted to be a branch-point splicing mutation by two prediction programs (SVM-BPfinder and Human Splicing Finder). c.545+1G>A was found in F6, which was an obvious splice donor site mutation. c.545+1G>A was reported in ExAC with a very low allele frequency (0.001902%) and was absent in ESP6500. Several programs (ASSP, NetGene2, Human Splicing Finder, SplicePort and NNSPLICE) were utilized to predict its effect; however, each program generated a number of different results.

**Fig 2:**
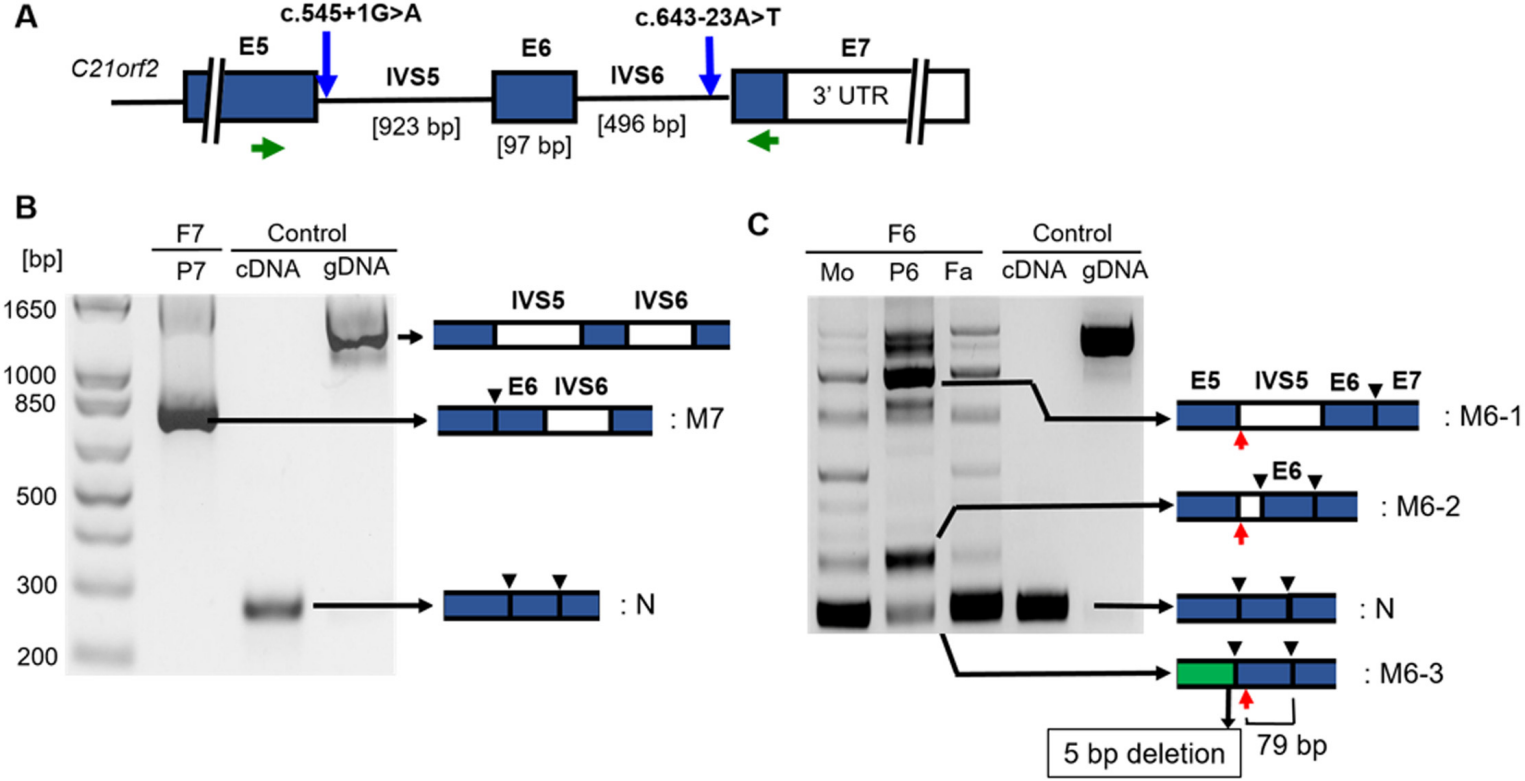
Analysis of the splicing mutations. (A) A schematic of the local genomic structure of *C21orf2*. Positions of the splicing donor site mutation (c.545+1G>A) in F6 and the branch-point mutation (c.643-23A>T) in F1 and F7 are indicated by blue arrows. E: exon, IVS: intron, Green arrows: positions of RT-PCR primers. B) RT-PCR analysis for c.643-23A>T. Intron 6 was not spliced in the mutant transcript (M7), which had a frame shift with the elongated reading frame. N: normal transcript. Black arrowhead: splicing junction in specific transcript. C) RT-PCR analysis for c.545+1G>A. In the family members (F6), aberrant bands with various sizes (M6-1~3) were obtained. Sanger-sequencing revealed that M6-3, an apparently normal size band in the patient (P6) represented a miss-spliced mutant which lost 5 bp in the end of exon 5. Red arrow: position of the stop codon. In M6-1 and 3, the new stop codons are more than 55 bp upstream of the last splicing junctions. In M6-2, the new stop codon is in the 3^rd^ last exon. Therefore, all these transcripts are considered to receive nonsense-mediated mRNA decay. Mo: the mother; Fa: the father.

A primer set spanning from exon 5 to exon 7 (Fig 2A) was used to check the effects of the intronic variants in mRNAs of P6 and P7. RT-PCR of P7 showed a single band with a markedly increased size in comparison to control subjects (Fig 2B). Direct sequencing of the PCR product identified that entire intron 6 remained in the mutant mRNA, which led to a frame shift and produced an elongated protein (p.N215Vfs*259) without the C-terminal conserved region.

RT-PCR of P6 generated a series of bands (Fig 2C) in the same conditions validated by control cDNA samples and P7. PCR products of P6 were cloned and sequenced. Sequencing results showed that several cryptic donor sites in exon 5 and intron 5 were utilized in the mutant genome, and were responsible for the multiple bands in the RT-PCR (Fig 2C). Interestingly, an amplicon from the patient’s cDNA, which appeared to have the same molecular size as the PCR product of normal control individuals, was demonstrated to have an abnormal sequence. Sanger sequencing showed that this band represented a transcript with 5-bp deletion generated by the splicing that employed the GC dinucleotide 5-bp upstream of the constitutive donor site as the new splice donor site. The deletion would cause a frame-shift and generate a truncated protein p.A181Qfs*6. Sequencing of other bands specific to the patient showed that they were composed by transcripts with partial (5’ end) or entire intron 5 retention. Because a stop codon was formed immediately after the junction of exon 5 and remained intron 5, all these transcripts are predicted to generate a truncated protein, p.S183*. Therefore, all the mutant transcripts in P6 are predicted to generate truncated proteins without the C-terminal conserved domain when transcribed. However, because the positions of the new stop codons produced by the aberrant splicing mutations were more than 55 bp upstream of the last splicing junction (Fig 2C), those transcripts would receive nonsense mediated mRNA decay [9,10].

### Patients without *C21orf2* mutation

**Fig 3:**
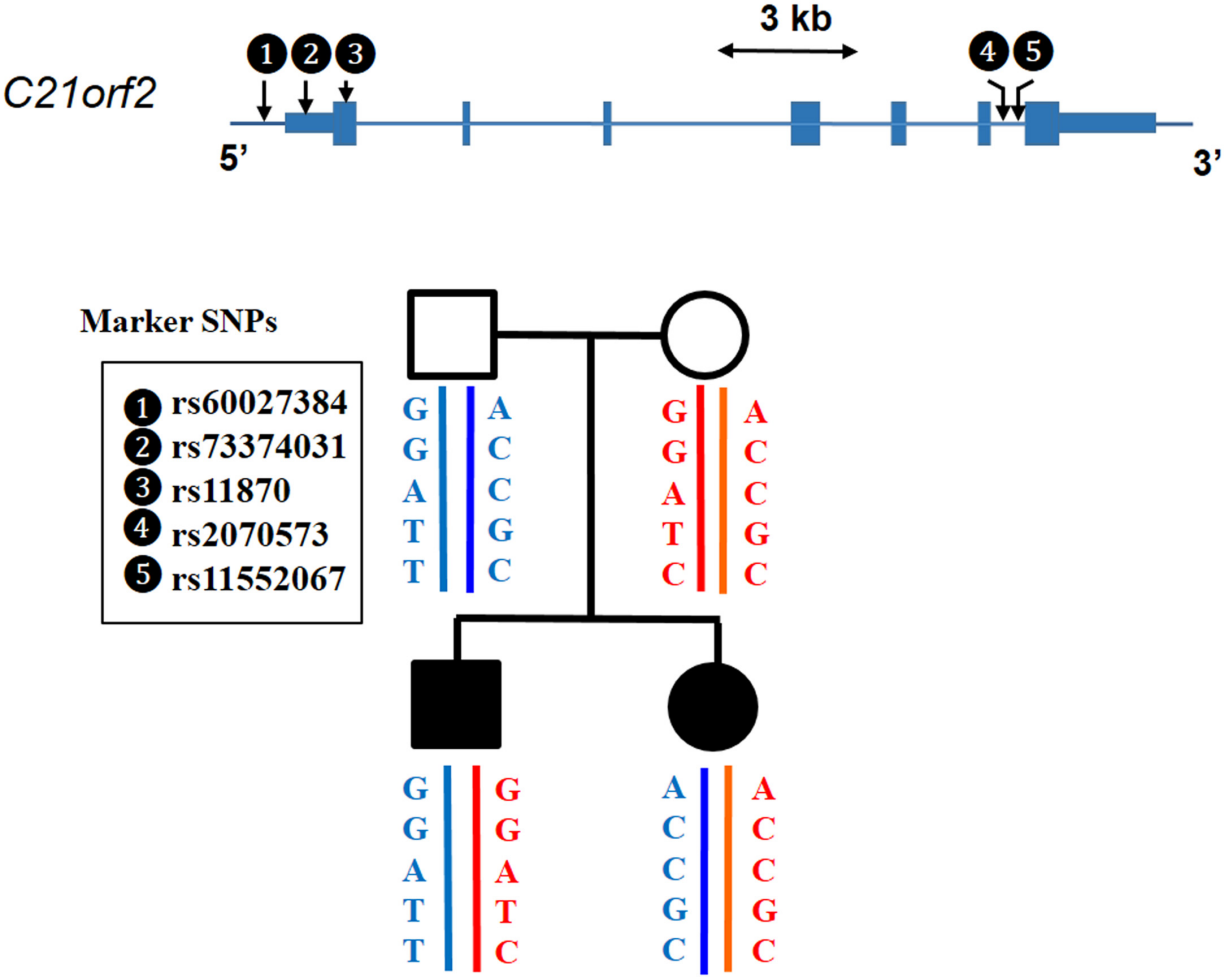
A haplotype analysis of C*21orf2* in family F3. The sib patients inherited different C*21orf2* haplotypes from the parents, respectively, which ruled out *C21orf2* as a disease gene in this family.

In families F2, F3 and F4, no candidate mutation was identified in coding region of *C21orf2* from exome sequencing results. We Sanger-sequenced 5’ and 3’ UTRs of *C21orf2* which were not included in the exome captured regions, as well as the exons with lower coverage in exome sequencing; however, no mutations were found. We then examined the *C21orf2* haplotypes in both affected siblings and their parents in F3. The two affected children inherited different alleles from their parents, respectively (Fig 3). Therefore, *C21orf2* could be excluded as a disease gene in F3.

In families F2 and F4, RT-PCR of *C21orf2* CDS showed normal band size and sequence in the patients, which excluded the possibility of exon-scale insertion/deletion; both patients were heterozygous for at least six SNPs within *C21orf2*, which excluded gene-scale insertion/deletion. Therefore, *C21orf2* is also not likely to be the disease gene for F2 and F4.

### Function of *C21orf2* in chondrocyte

**Fig 4:**
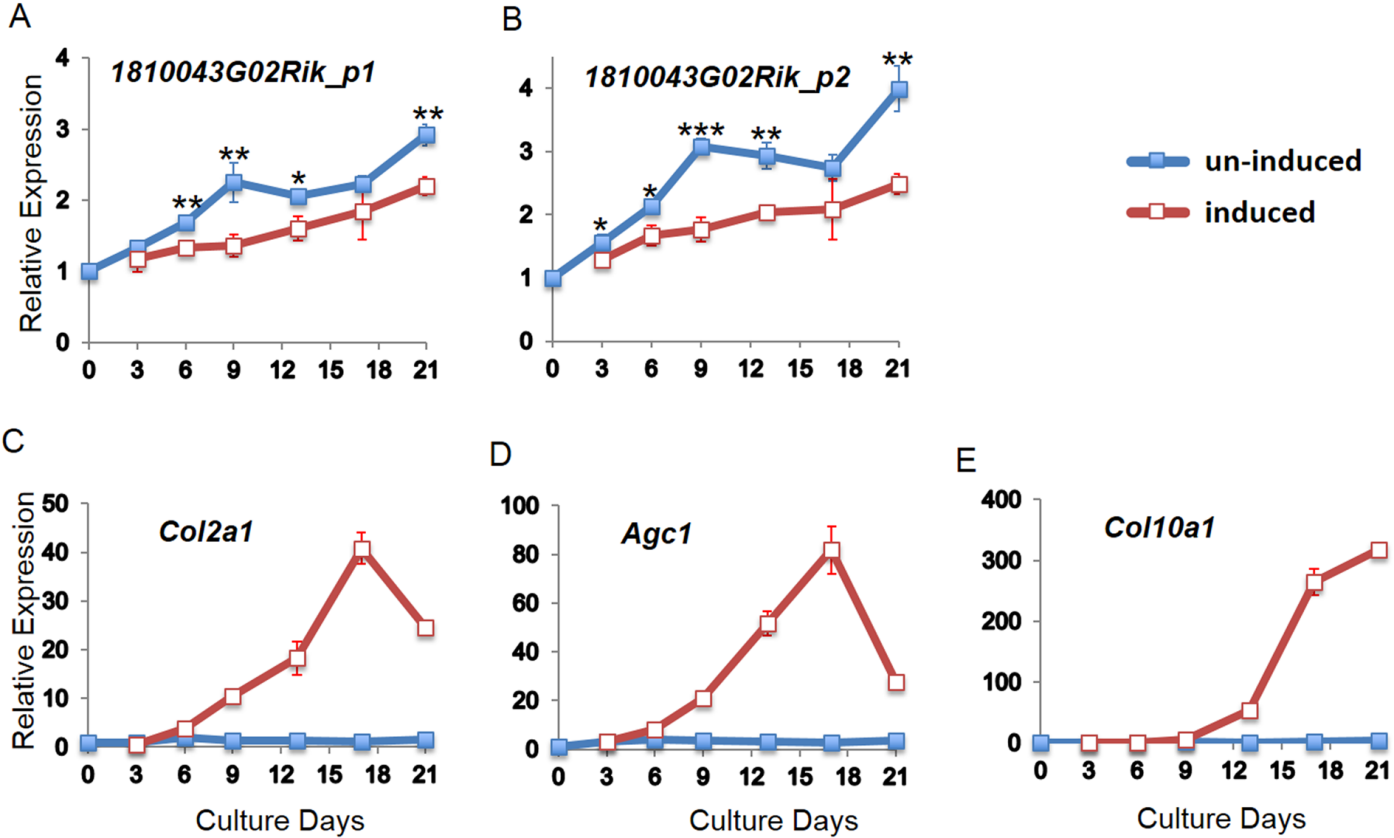
*C21orf2* expression during chondrocyte induction. Relative mRNA expression of mouse *C21orf2* (*1810043G02Rik)* in induced (red lines) and un-induced (blue lines) ATDC5 cells. (A-B) The expression of *1810043G02Rik* measured by real-time PCR using two primer sets; (C-E) Expression of chondrocyte marker genes (*Col2a1*, *Agc1* and *Col10a1*), indicating the differentiation of induced ATDC5 cell to chondrocyte. All the expression values were presented relatively to the ones of day 0, which was set as 1. *: P< 0.05, **: P< 0.01, ***: P< 0.001; induced versus un-induced by t-test. n = 3.

Skeletal phenotypes of axial SMD suggest *C21orf2* plays an important role in skeletal formation and development. To gain insight into the role of *C21orf2* in cartilage development, we examined *1810043G02Rik* (mouse homologue of *C21orf2*) mRNA expression during the differentiation process to chondrocyte using ATDC5 cell, an *in vitro* mouse model of chondrocyte differentiation [11]. While the expression of cartilage marker genes (*Col2a1*, *Agc1* and *Col10a1*) was increased by the cartilage induction, *1810043G02Rik* expression was continuously suppressed during cartilage differentiation (Fig 4).

**Fig 5:**
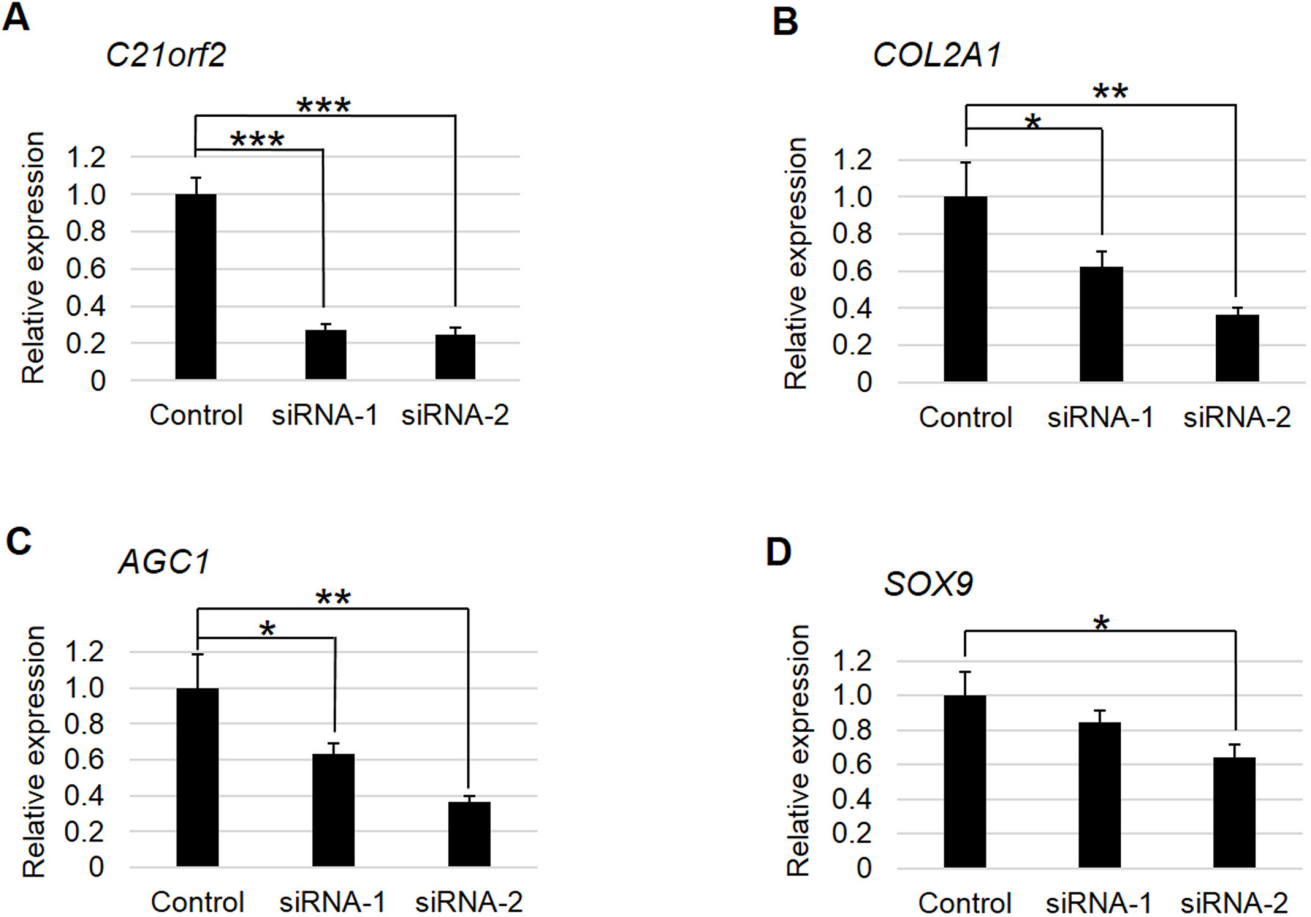
Effects of siRNAs for *C21orf2* on chondrocyte marker genes in OUMS-27 cell. (A) *C21orf2* was significantly knocked-down by both siRNAs (siRNA-1 and 2). (B-D) mRNA expression of chondrocyte differentiation marker genes. The expression of the marker genes decreased when *C21orf2* was knocked-down. *: P< 0.05, **: P< 0.01, ***: P< 0.001; versus control by t-test. n = 3.

We then examined *C21orf2* function by transfecting *C21orf2* siRNAs to OUMS-27, a human cell lines derived from chondrosarcoma with chondrocytic characteristics. Knock-down of *C21orf2* caused significant decreases in expression of chondrocyte marker genes (Fig 5). These results suggested that *C21orf2* is necessary for maintenance of the differentiated chondrocyte phenotype. Further studies are necessary to clarify the role of *C21orf2* in cartilage.

### Subcellular localization of *C21orf2* in retina

**Fig 6:**
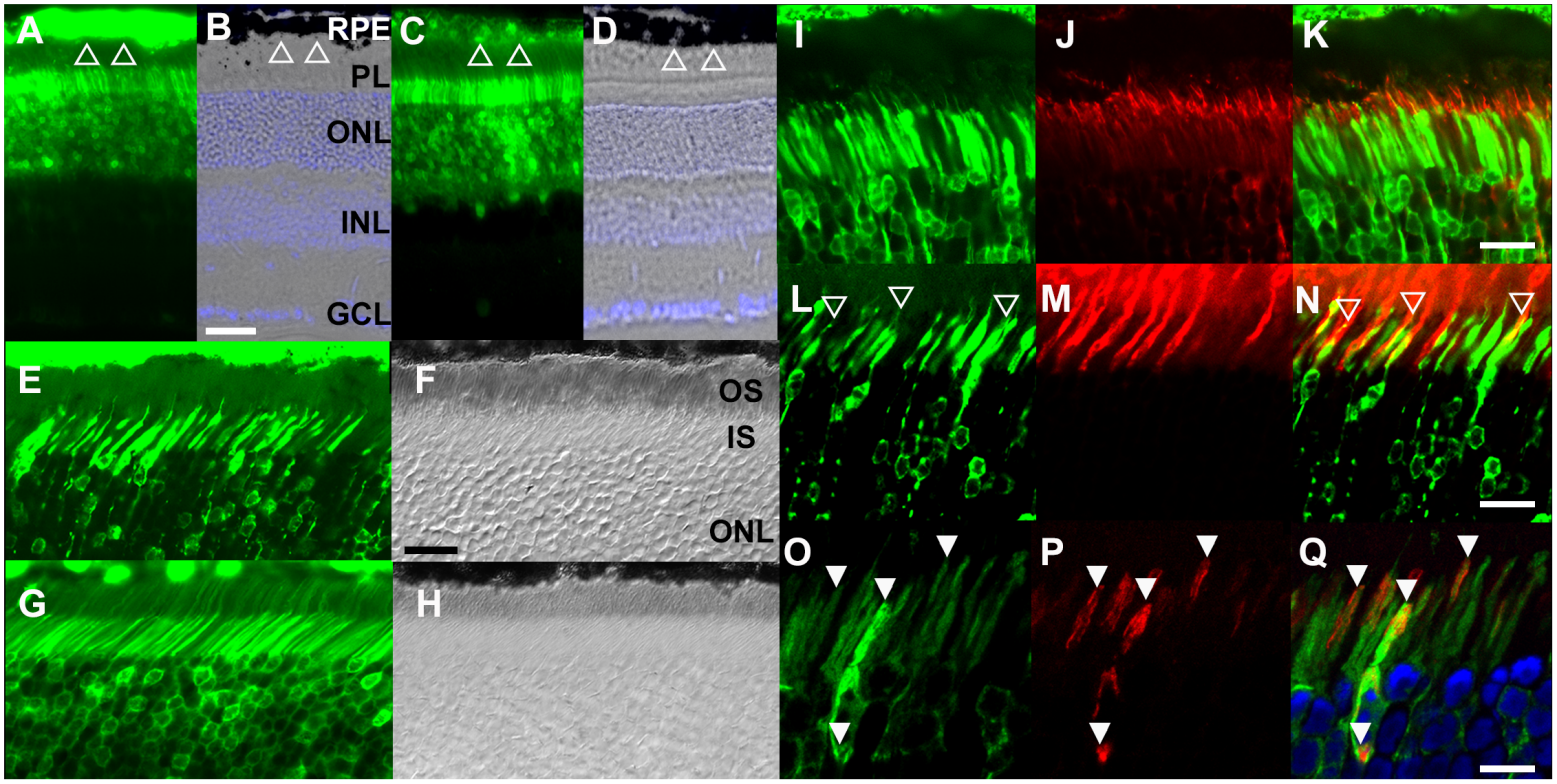
*C21orf2* localized to the connecting cilium of the rod and cone photoreceptors. (A-D) Expression of EGFP driven by CMV-promoter or *C21orf2*-promoter. When driven by the ubiquitous CMV-promoter, EGFP showed stronger expression in the retinal pigment epithelium (RPE; Open triangle) than in the photoreceptors (A, B). When driven by the *C21orf2*-promoter, EGFP is expressed more prominently in photoreceptors than in RPE (C, D). (E-H) AAV8-mediated expression of EGFP fusion protein. The C21orf2-EGFP fusion protein was not detected in the outer segments (OS; E, F), while EGFP was present in the OS in the control (G, H). (I-K) C21orf2 localized to the connecting cilium (red; stained with anti-acetylated-tubulin antibodies). (L-N) Association of C21orf2 to the connecting cilium, but not to the surrounding OS structure in cone photoreceptors. C21orf2-EGFP fusion protein remains localized to the cilia (open arrowheads) inside the PNA-positive cone OS (red). (O-Q) Lack of spatial association between C21orf2 and mitochondria. Kusabira Orange-tagged mitochondria (red). RPE, retinal pigment epithelium; PL, photoreceptor layer; ONL, outer nuclear layer; INL, inner nuclear layer; GCL, ganglion cell layer; OS, outer segment; IS inner segment. Scales bars: 50 μm (B), 30 μm (H, K, N) and 15 μm (Q).

Axial SMD is characterized by retinopathy. Our RT-PCR confirmed the expression of *C21orf2* in retina (S3 Fig); however, retina is a multi-layer tissue composed of highly differentiated cells with diverse functions. To gain further insight into the role of *C21orf2* in retina, we investigated localization of *C21orf2 in vivo* by injecting the designed AAV vectors into the mouse retina. We generated the construct with the EGFP reporter gene fused after the *C21orf2* promoter and compared its transcription activity with that fused after the CMV promoter. When driven by the CMV promoter, the reporter gene expression was by far the strongest in the retinal pigment epithelium (RPE) (Fig 6A and 6B), as previously reported [12]. In contrast, when driven by the *C21orf2* promoter, the most prominently expressed region shifted to photoreceptors and a subset of cells at the outer limits of the inner nuclear layer (INL); the expression in RPE was limited (Fig 6C and 6D). These results are consistent with *C21orf2* expression in the photoreceptors.

To determine the subcellular localization of C21orf2 in photoreceptor cells, we generated a vector containing *C21orf2-EGFP* fusion construct and sub-retinally injected to mouse eyes. The result showed that C21orf2-EGFP fusion protein was present in the inner segments, but absent in the neighboring outer segments (Fig 6E–6H). At the junction of the two segments, the fusion protein exhibited a cilia-like structure, and co-stained with acetylated tubulin, a cilia marker [13] (Fig 6I–6K). Furthermore, we observed that the C21orf2-EGFP fusion protein extended into the PNA (peanut agglutinin)-positive outer segments of cone photoreceptors (Fig 6L–6N), which appeared strictly confined to the cilia without dispersing into the surrounding outer segment structures. C21orf2 protein is reported to localize in mitochondria in EBV-transformed B cells [14]. An area at the distal compartment of the inner segments is known to be enriched with mitochondria [15]. We stained mitochondria by Kusabira-Orange fused with mitochondria localizing signal and found that subcellular distribution of the C21orf2-EGFP fusion protein was complementary to that of mitochondria (Fig 6O–6Q). Taken together, these results indicate that in the photoreceptors, C21orf2 protein is localized at the connecting cilia. It was reported that ciliary structure bridges the inner and outer segments in photoreceptor cells [16,17], and the majority of the syndromic retinal dystrophy are associated with the diseased ciliary structure [18].

### Genotype-phenotype relationship

While we were preparing the manuscript, *C21orf2* mutations have been identified in some patients diagnosed as Jeune syndrome [6]. Jeune syndrome belongs to a group of ciliopathies with major skeletal involvement (skeletal ciliopathy) [19] and is characterized by constricted thoracic cage, short ribs, shortened tubular bones, and a 'trident' appearance of the acetabular roof. Cone shaped epiphyses and handlebar clavicles are often observed. Polydactyly is found in some cases [20,21]. Jeune syndrome is a clinically and genetically heterogeneous group of disorders. Seven causal genes are listed in the recent revision of the nosology and classification of genetic skeletal disorders [22]. It is differentiated from axial SMD by 1) severe brachydactyly, 2) absence of spondylar dysplasia, and 3) absence of lacy iliac crest.

Combining a whole-genome siRNA-based reverse genetics screen and exome sequencing, Wheway *et al*. identified *C21orf2* as a cause of Jeune syndrome and placed C21orf2 within key ciliopathy-associated protein modules [6]. They also showed *c21orf2* localisation to photoreceptors. Their patients included homozygotes of c.545+1C>T and c.218G>C. The patient with c.545+1C>T was previously reported [7] and is confirmed to have no skeletal abnormality, while our patient (P6) with the same homozygous mutation had severe skeletal dysplasia. The skeletal phenotypes of the family members with the c.218G>C mutation were similar to our axial SMD patients (P8-1~3, P9) with the same mutation, except for the absence of typical thoracic deformity in 3/5 members. The mutation has been functionally evaluated by siRNA knock down-rescue and found to be hypomorphic [6]. All patients in the paper except one have childhood onset cone-rod dystrophy like our axial SMD patients. Thus, the effects of *C21orf2* mutations are relatively predictable in retina, but highly variable in skeleton.

### Conclusion

In conclusion, we have identified *C21orf2* as the disease gene for axial SMD, a unique disease affecting the skeleton and retina. Genetic heterogeneity definitely exists for axial SMD; other gene(s), most probably cilia-related gene(s) could also cause axial SMD phenotype. We have added axial SMD to the rapidly growing list of skeletal ciliopathy with retinal manifestations. Also, we have presented another example of the power and advantage of the whole exome sequencing approach for a group of complex diseases like ciliopathy that has a wide clinical variability and genetic heterogeneity. Further studies would be necessary to clarify the detailed function of *C21orf2* in skeletal development and retinal function.

## Materials and Methods

### Nucleic acid preparation

Written informed consents were obtained from all the participants; for the minors included in the study, informed consents were obtained from their parents or guardians. This study is approved by the Ethics Committee of RIKEN center for Integrative Medical Sciences (approval number: H16–40).

Genomic DNAs were extracted from peripheral blood with QIAamp DNA Blood Midi Kit (Qiagen) by following the manufacturer’s protocol.

Total RNAs of families F2, F4, F6 and F7 were available. For patients P2 and P4, total RNAs were extracted from lymphoblastoid cells by using ISOGEN (Nippon Gene) and QIAamp RNA Blood Mini Kit column (Qiagen). For P6 and both his parents, peripheral blood samples were collected in PAXgene Blood RNA Tubes (Qiagen), and then total RNAs were extracted by using PAXgene Blood RNA Kit (Qiagen). For P7, total RNA was extracted from peripheral blood by using TRIzol Reagent (Life Technologies) and QIAamp RNA Blood Mini Kit column (Qiagen).

DNA and RNA concentrations were measured on NanoVue Spectrophotometer (GE Healthcare) for reverse transcription or PCR or Qubit 2.0 Fluorometer (Life Technologies) for whole exome sequencing. Total RNA was reverse-transcribed to cDNA by using High Capacity cDNA Reverse Transcription Kit (Life Technologies) and random hexamer primers (Life Technologies).

cDNA from various tissues (cartilage, bone, disc, retina, brain, heart, lung, liver, spleen, kidney and skeletal muscle) (ClonTech) and cell lines (MG63, SAOS2, OUMS-27, HCS2/8, SW1353, HeLa, HEK293, and HuH-7) were used as normal controls and for validation the gene structure and expression of *C21orf2*.

### Exome sequencing and variation calling

Exome sequencing was performed on 10 patients as previously described [23,24]. Briefly, DNA (3 μg) was sheared by an S2 system (Covaris) and processed by SureSelect Human All Exon kit or SureSelectXT Human All Exon V5 (Agilent Technologies). Captured DNAs were sequenced by using HiSeq 2000 (Illumina) with 101-bp pair-end reads with 7 indices. Image analysis and base calling were performed by using HCS, RTA and CASAVA softwares (Illumina). Reads were mapped to the reference human genome (hg19) by Novoalign-3.00.02 or 3.02.04. Aligned reads were processed by Picard to remove PCR duplicate. Variants were called by GATK (v1.6–5 or v2.7–4) [25] following the recommended workflow [26][27], and annotated by ANNOVAR [28].

### PCR, RT-PCR and Sanger sequencing

Several primer sets were designed to: 1) validate the mutations identified in exome sequencing; 2) detect mutations directly; 3) validate the splicing isoforms; or 4) confirm the effects of splicing mutation. Primers sequences and PCR conditions were available on request. Sanger sequencing was performed on a 3730 DNA analyzer (Life Technologies). PCR products were cloned when necessary by using TOPO TA Cloning Kit (Life Technologies) and One Shot TOP10 Chemically Competent *E*. *coli* (Life Technologies). Sequencher (ver. 4.7, Gene Codes) and Mutation Surveyor (ver. 4.0.6, SoftGenetics) were used for aligning sequencing chromatographs to reference sequences and mutation detection.

### Population frequency

One hundred Turkish and 606 Korean individuals were used as population controls for each ethnic group with informed consent. SNPs of interest were genotyped by invader assay [29] and frequencies of specific genotypes were calculated.

### *In silico* analysis

For a sequence conservation analysis, protein sequences of human (C21orf2, NP_004919.1), chimpanzee (C21H21orf2, XP_514938.2), cattle (C1H21orf2, NP_001069249.1), mouse (1810043G02Rik, NP_080707.2), rat (RGD1309594, NP_001008352.1), chicken (C9H21ORF2, NP_001006544.1), were downloaded from Genbank and aligned in ClustalX (ver. 2.1)[30].

Domain architecture was predicted by InterPro [31]. Wild-type protein sequence of human C21orf2 (NP_004919.1) and the mutant protein sequences with missense mutations found in this study were submitted to I-TASSER [32,33] for 3D structure prediction.

The effects of missense variations were annotated by SIFT [34], PolyPhen2 [35] and MutationTaster [36], through the pipeline of ANNOVAR. For the prediction on splicing mutations, genomic sequence of intron 6 of *C21orf2* was submitted to SVM-BPfinder [37] and Human Splicing Finder [38] for prediction of the branch-point. Genomic sequence from exon 6 to exon 7 of *C21orf2* was submitted to ASSP [39], NetGene2 [40], Human Splicing Finder [38], SplicePort[41], and NNSPLICE [42] for prediction of candidate splicing donor sites.

### Cell culture and gene expression assay

ATDC5 cells (RIKEN) were cultured and induced for differentiation into chondrocyte as previously described [43]. RNAs from the induced cells were extracted on day 0 (before induction) and on days 3, 6, 9, 13, 17, and 21 after induction; RNAs from corresponding non-induced cells (cultured in the same condition) was also extracted as controls. The expression of *1810043G02Rik*, the mouse homologue of *C21orf2* was measured by real-time RT-PCR on a StepOne realtime PCR system (Life Technologies). Two primer sets of *1810043G02Rik* were utilized for confirmation. Expression of chondrocyte differentiation marker genes, *Col2a1*, *Agc1* and *Col10a1*, were also measured by real-time RT-PCR. *Ppia* was used as reference gene[44]. All primer sequences and PCR conditions are available on request. Relative expression value was defined as a ratio of quantities of *C21orf2* or marker genes divided by the corresponding quantity of *Ppia*. T-test was performed between relative expression values of induced cell and un-induced cell at a given culture time.

OUMS-27 cells were cultured for knock-down experiments. siRNAs for *C21orf2* were synthesized (Life Technologies) against the following target sequences:

5’-AGCUGCACAGCGUGCGCAAGCUCAA-3’

5’-GCACUGAGUGAGGGAGAGGAGAUCA-3’

Stealth RNAi^™^ siRNA Negative Control Hi GC (Life Technologies) was used as a negative control. siRNAs were transfected into OUMS-27 cell on a 4D-Nucleofector System (Lonza), following the recommended protocol for OUMS-27, with an adaption of the transfection concentration of siRNA to 600 nM. Cells were harvested 48 hours after transfection. RNA was extracted and reverse transcribed immediately. Real-time PCR was performed to check the expression level of *C21orf2* and marker genes of chondrocyte differentiation.

### Histological assessment for *C21orf2* expression of in retina

The following four vectors were generated for investigating the expression of C21orf2 in retina:

1. rAAV2/8.CMV.EGFP: CMV promoter-driven-EGFP (Enhanced green fluorescent protein) was subcloned into a pAAV-MCS Promoterless Expression Vector (Cell Biolabs).
2. rAAV2/8.hC21orf2.EGFP: EGFP driven by the *C21orf2* promoter region of (1954 bp immediately upstream of the initiation codon of NM_004928.2) was subcloned into a pAAV-MCS Promoterless Expression Vector.
3. *C21orf2* CDS (NM_004928.2) was fused with EGFP cDNA in-frame. The fusion construct was subcloned into a pAAV-MCS vector (Agilent Technologies).
4. Mitochondria localizing signal (Cytochrome c oxidase polypeptide IV from Saccharomyces) was fused with Kusabira-Orange (KO) cDNA (MBL). The fusion construct was subcloned into pAAV-MCS vector.

AAV2/8 containing the reporter constructs described above were generated and purified as described previously [45]. AAV2/8 containing CMV promoter driven EGFP cDNA construct served as the control. Each virus (1 x 10^12^ gc/ml) was double injected (2 μl/ injection) into both the dorsal and the ventral sub-retinal space of a 6 weeks-old C57BL6 mouse (Japan SLC). The injected eyes were collected one week later, fixed in 4% paraformaldehyde, embedded in OCT compound (Sakura Finetek), and sectioned using a cryostat (model CM3050, Leica). In some cases, the section was further blocked with 5% goat serum for 30 min, incubated with anti-acetylated tubulin antibodies (T7451, 1: 1000, Sigma-Aldrich, St. Louis, MO) for 1 h, and stained with a second antibodies (anti-mouse Alexa Fluo 568, Life Techonologies), Rhodamine-conjugated peanut agglutinin (PNA; Vector Laboratories) or 4',6-diamidino-2-phenylindole (DAPI; Vector Laboratories) for additional 45 min.

### *In silico* resources

The URLs for data presented herein are as follows:

Human Gene Mutation Database (HGMD), https://portal.biobase-international.com/hgmd/pro/start.php

Reference Sequence (RefSeq) database, http://www.ncbi.nlm.nih.gov/refseq/

FANTOM 5, http://fantom.gsc.riken.jp/5/

MGI Gene Expression Database, http://www.informatics.jax.org/expression.shtml

HomoloGene,http://www.ncbi.nlm.nih.gov/homologene

Basic Local Alignment Search Tool (BLAST), http://blast.ncbi.nlm.nih.gov/Blast.cgi

ClustalX, http://www.clustal.org/clustal2/

ESP6500, http://evs.gs.washington.edu/EVS/

ExAC, http://exac.broadinstitute.org

SIFT, http://sift.jcvi.org

PolyPhen-2, http://genetics.bwh.harvard.edu/pph2/

MutationTaster, http://www.mutationtaster.org

I-tasser, http://zhanglab.ccmb.med.umich.edu/I-TASSER/

SVM-BPfinder, http://regulatorygenomics.upf.edu/Software/SVM_BP/

Human Splicing Finder, http://www.umd.be/HSF3/

Alternative Splice Site Predictor (ASSP), http://wangcomputing.com/assp/

NetGene2, http://www.cbs.dtu.dk/services/NetGene2/

SplicePort, http://spliceport.cbcb.umd.edu

NNSPLICE ver. 0.9, http://www.fruitfly.org/seq_tools/splice.html

## Supporting Information

S1 Fig Clinical features of axial spondylometaphyseal dysplasia (axial SMD) patients with *C21orf2* mutations. (PDF)

S2 Fig Axial SMD pedigrees in this study. (PDF)

S3 Fig *C21orf2* expression in human. (PDF)

S4 Fig *C21orf2* is highly conserved among diverse species. (PDF)

S5 Fig Two missense *C21orf2* mutations in Family 5. (PDF)

S1 Table Summary of the exome sequencing performance. (PDF)
