# Supplementary material for: Axial Spondylometaphyseal Dysplasia Is Caused by C21orf2 Mutations
Source: PLoS One. 2016 Mar 14;11(3):e0150555. doi: 10.1371/journal.pone.0150555 (PMC4790905; doi:10.1371/journal.pone.0150555)
Supplement: S1 Fig — (PDF) [file pone.0150555.s001.pdf]

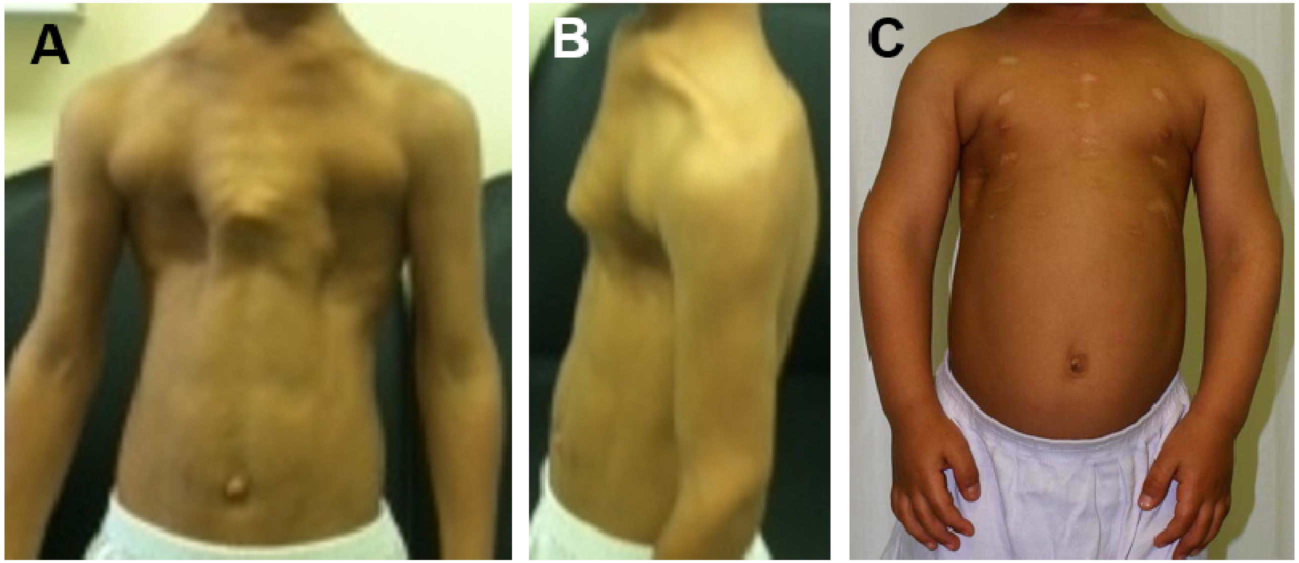

S1 Fig. Clinical features of axial spondylometaphyseal dysplasia (axial SMD) patients with *C21orf2* mutations.

(A), (B) P7, 9-year-old boy. Very narrow chest, severe Harrison sulci with pectus carinatum. (C) P1-2, 5-year-old boy. Mild chest deformity. Both are homozygotes of the same *C21orf2* mutation, c.643-23A>T.
