# Supplementary figures and images for: Axial Spondylometaphyseal Dysplasia Is Caused by C21orf2 Mutations
Source: PLoS One. 2016 Mar 14;11(3):e0150555. doi: 10.1371/journal.pone.0150555 (PMC4790905; doi:10.1371/journal.pone.0150555)

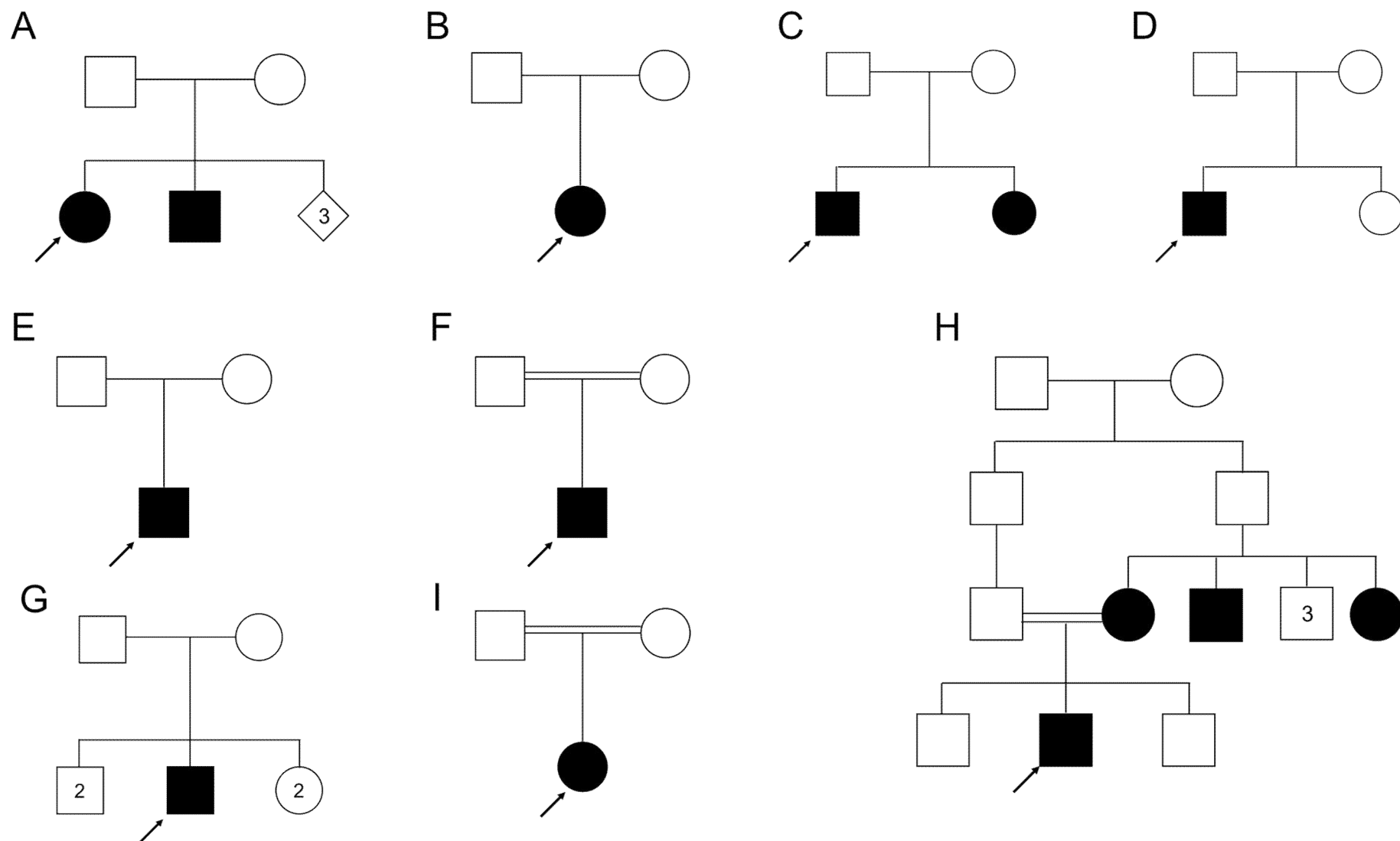

**S2 Fig. Axial SMD pedigrees in this study.** (A) F1, (B) F2, (C) F3, (D) F4, (E) F5, (F) F6, (G) F7, (H) F8, (I) F9.

Supplement: S2 Fig — (PDF) [file pone.0150555.s002.pdf]
