# Supplementary material for: Axial Spondylometaphyseal Dysplasia Is Caused by C21orf2 Mutations
Source: PLoS One. 2016 Mar 14;11(3):e0150555. doi: 10.1371/journal.pone.0150555 (PMC4790905; doi:10.1371/journal.pone.0150555)
Supplement: S3 Fig — (PDF) [file pone.0150555.s003.pdf]

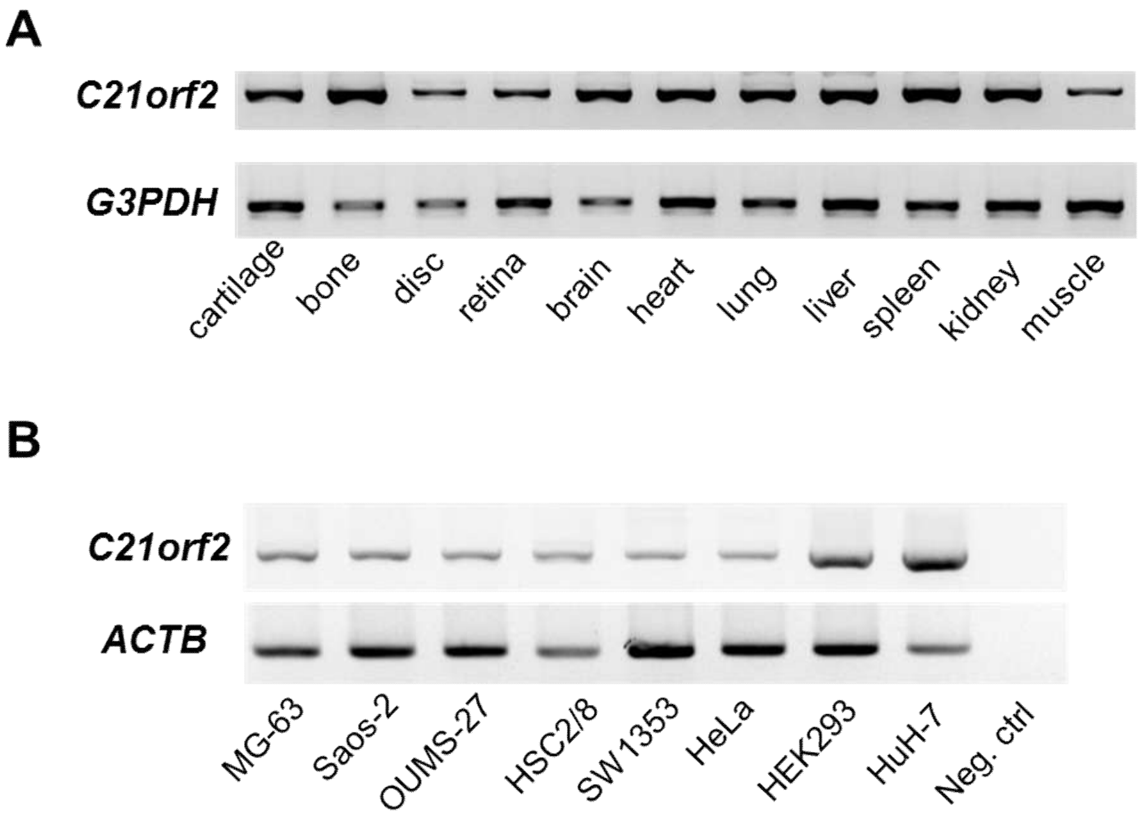

S3 Fig. *C21orf2* expression in human.

RT-PCR for (A) various tissues and (B) cell lines. *C21orf2* mRNA is ubiquitous expressed, including in tissues probably affected in axial SMD (cartilage, bone, vertebral disc and retina).
