# Supplementary material for: Axial Spondylometaphyseal Dysplasia Is Caused by C21orf2 Mutations
Source: PLoS One. 2016 Mar 14;11(3):e0150555. doi: 10.1371/journal.pone.0150555 (PMC4790905; doi:10.1371/journal.pone.0150555)
Supplement: S4 Fig — (PDF) [file pone.0150555.s004.pdf]

A

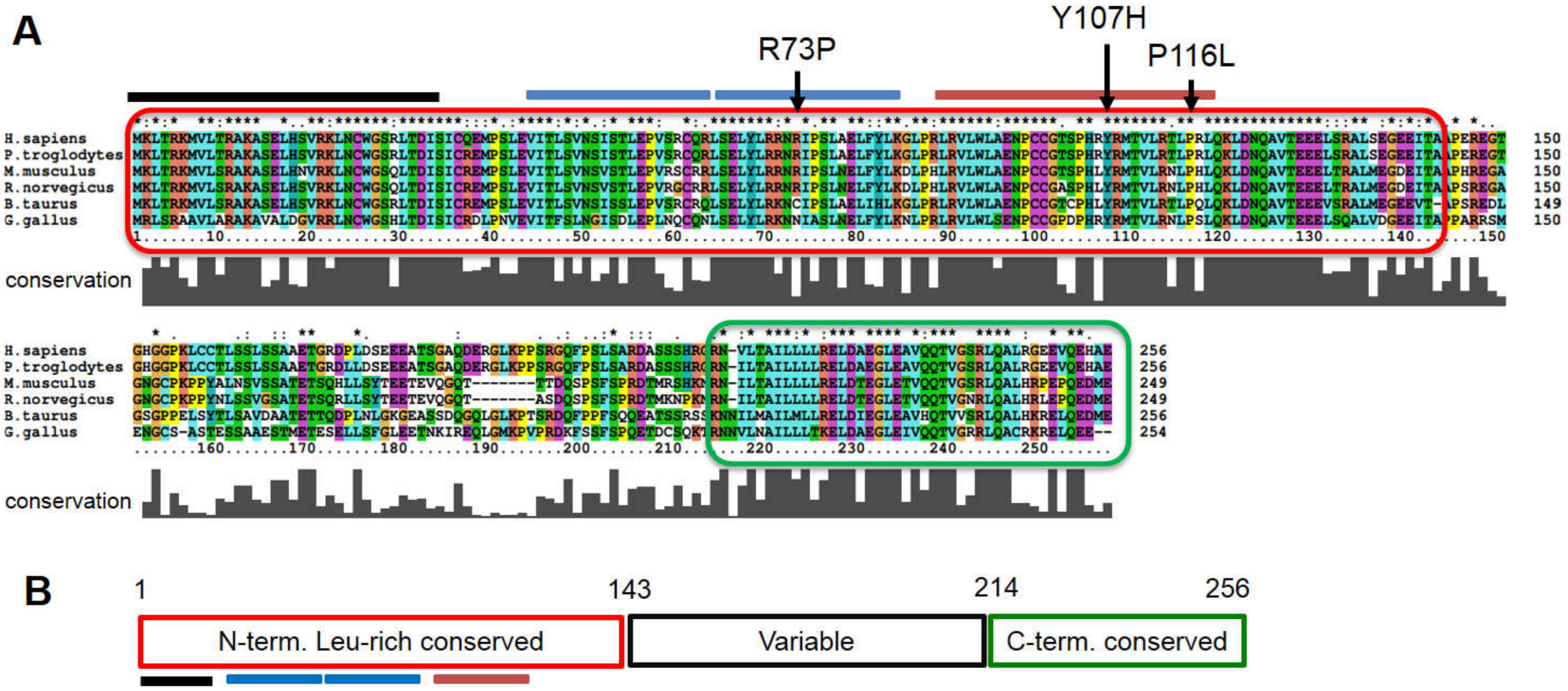

C

Amino acid conservation

Species	Identity (%)*	Length (aa)
H. sapiens		256
P. troglodytes	99	256
B. taurus	74	256
M. musculus	73	249
G. gallus	61	254
X. tropicalis	51	212
D. rerio	55	365

* by BLASTp

S4 Fig. *C21orf2* is highly conserved among diverse species.

(A) ClustalX 2.1 multiple sequence alignment. There are 2 conserved regions: N-terminal region (red box) and C-terminal region (green box). Black bar: predicted mitochondria localization signal. There are two tandem LRR_4 domains (blue bars) followed by an LRRcap domain (brown bar). Arrow heads: position of missense mutations. (B) A schematic structure of human C21orf2 protein. (C) Amino acid conservation among different species.
