# Supplementary material for: Axial Spondylometaphyseal Dysplasia Is Caused by C21orf2 Mutations
Source: PLoS One. 2016 Mar 14;11(3):e0150555. doi: 10.1371/journal.pone.0150555 (PMC4790905; doi:10.1371/journal.pone.0150555)
Supplement: S5 Fig — (PDF) [file pone.0150555.s005.pdf]

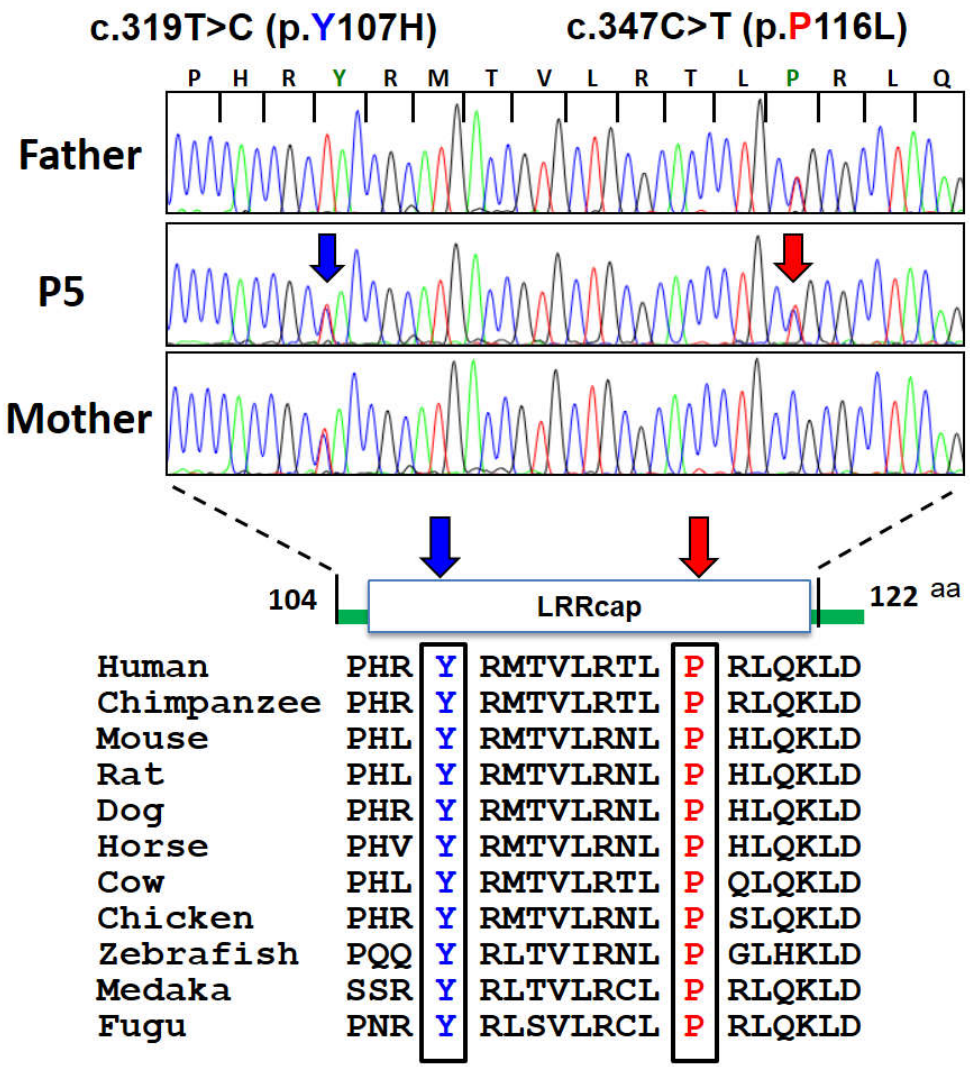

S5 Fig. Two missense *C21orf2* mutations in Family 5.

The Korean patient is a compound heterozygote for the mutations. The mutated amino acids are evolutionally highly conserved. LRRcap: leucine-rich repeat cap domain (A motif occurring C-terminal to LRRs in typical LRR-containing proteins).
