# Supplementary material for: Axial Spondylometaphyseal Dysplasia Is Caused by C21orf2 Mutations
Source: PLoS One. 2016 Mar 14;11(3):e0150555. doi: 10.1371/journal.pone.0150555 (PMC4790905; doi:10.1371/journal.pone.0150555)
Supplement: S1 Table — (PDF) [file pone.0150555.s006.pdf]

S1 Table. Summary of the exome sequencing performance

Family ID	Subject ID	Coverage			
		No. of bases	Average depth	% bases > 10x	% bases > 20x
F1	P2	3,237,878,527	96.73	96.2	93.2
F2	P3	7,322,411,597	218.76	97.2	96.5
F3	P4	2,534,981,177	75.73	91.5	83.9
F3	P5	6,835,737,251	204.22	97.2	96.3
F4	P6	6,200,400,067	185.24	97.2	96.3
F5	P7	6,242,207,776	186.49	97	95.7
F6	P8	3,653,285,254	109.14	96.3	93.7
F7	P9	3,857,929,421	115.26	96.5	94.1
F8	P10	3,445,155,985	102.93	96.4	93.6
F8	P11	3,853,068,512	115.11	96.6	94.5
F8	P12	2,978,147,369	88.97	96.2	92.8
